# Ascorbic acid enhances adipogenesis of 3T3-L1 murine preadipocyte through differential expression of collagens

**DOI:** 10.1186/1476-511X-12-182

**Published:** 2013-12-11

**Authors:** Byoungjae Kim, Kyung Min Choi, Hong Soon Yim, Min-Goo Lee

**Affiliations:** 1 Department of Physiology, Korea University College of Medicine, Seoul 136-705, Republic of Korea

**Keywords:** Ascorbic acid, adipogenesis, 3T3-L1, collagens, differential expression

## Abstract

**Background:**

Adipogenesis from preadipocytes into mature adipocyte is precisely coordinated by transcription factors such as CCAAT-enhancer-binding proteins (C/EBPs) and peroxisome proliferator-activated receptor γ (PPARγ), cytokines, and hormones, which is accompanied by extracellular matrix remodeling. Besides anti-oxidant activity, ascorbic acid (ASC) is participating in collagen biosynthesis and increase production and processing of collagens. Moreover, several studies demonstrated that ASC enhanced differentiation from preadipocytes into mature adipocytes.

**Methods:**

The adipogenic effect of ascorbic acid was evaluated in chemical induced 3T3-L1 by Oil Red O staining. This effect was elucidated by immunoblotting which detected the expression level of collagens and transcription factors in adipogenesis. The immunocytochemical determination of type I collagen was performed in 3T3-L1 adipocyte to show the change of extracellular matrix during adipogenesis.

**Results:**

In this study, Oil Red O staining in 3T3-L1 preadipocytes was increased dose-dependently by addition of ASC. These ASC-treated adipocytes increased collagen processing of α1(I) and α1(V) and expressed α1(VI) and α2(VI) collagens differentially. ASC also stimulated expression of C/EBPα and PPARγ, which is preceded by collagen enhancement. In addition, inhibition of ASC activity by ethyl-3,4-dihydroxybenzoate showed reduction of lipid accumulation by removal of large lipid droplets, not by inhibition of lipid production. This observation went with loss of α1(I) deposition on adipocyte surface, increase of α1(V) and α2(VI) collagens and decrease of C/EBPs.

**Conclusion:**

Our findings imply that various actions of ASC on adipogenesis through differential collagen expression may provide diverse applications of ASC to adipose tissue technology.

## Introduction

Adipocyte differentiation from stem cells has two phases, determination and terminal differentiation. While the former is a commitment process from pluripotent stem cell to adipocyte lineage, the latter is a complex process that the preadipocytes develop into mature adipocyte, is coordinated by transcription factors such as C/EBPs and PPARγ, cytokines, and hormones and is commonly called adipogenesis [1,2].

In the process of adipogenesis, extracellular matrix (ECM) remodeling occurs for appropriate differentiation environment with diverse expression pattern of collagens, a major ECM component. As murine preadipocytes lose their fibroblastic characteristics, the transcriptional level of fibrillar collagen type I and III in stroma was decreased while the expression of type IV collagen in basement membrane was increased [3,4]. Yi et al. [5] also reported that type I collagen of 3T3-L1 preadipocytes was repressed transcriptionally during adipogenesis by decreased promoter activity. On the other hand, when the surface collagens in bovine intramuscular preadipocyte were detected by isotoped antibody, all major collagens (type I – type VI) were increased after adipogenic induction and maintained throughout adipogenesis [6]. In addition, scanning electron microscopy and immunohistochemistry showed that the surface of adipocytes differentiated from stromal vascular cells of mouse adipose tissue was stained by collagen type I, III, V, and VI forming fibrillar networks at the late phase of adipogenesis [7]. Among these collagens, a2(VI) is considered a marker of the preadipose state because its mRNA rises sharply after full confluence and then gradually decreases in murine TA1 preadipocytes [8,9].

Besides anti-oxidant activity, ascorbic acid (ASC) acts as a cofactor of the hydroxylating enzyme of proline and lysine residues in procollagen. In addition to stabilization of collagen triple helix, ASC regulates collagen synthesis, cell growth, and cell differentiation [10,11]. ASC has been demonstrated to enhance differentiation of stem cells into cardiac lineage [12,13], differentiation of epidermal keratinocytes into skin structure [14] and differentiation of stem cells into neurons by increasing the expression of genes involved in neurogenesis [15]. In adipogenesis, long acting derivative of ASC has stimulated lipid accumulation by increasing collagen synthesis in 3T3-L1 cells [16]. Recently, collagen production and its adipogenic effect have also been reported in bone marrow-derived mesenchymal stromal cells [17,18]. However, little is known about the effect of ASC-induced collagen synthesis on adipogenesis although a few studies about this effect on adipogenesis were performed using ethyl-3,4-dihydroxybenzoate (EDHB), a specific inhibitor of collagen synthesis [9,19].

Murine preadipocyte 3T3-L1 cells are commonly used for adipogenesis research because these cells can be homogeneously differentiated compared with other preadipocytes [20]. Herein, we adopted 3T3-L1 cell line as a tool for the study about differential expression of collagens by ASC and its effect on adipogenesis with increased expression of transcription factors. In addition to direct effect of ASC, we investigated how the inhibitory action of EDHB affect the expression pattern of collagens and transcription factors in lipid accumulation for further information about ASC-induced adipogenic effect.

## Results

### ASC stimulates accumulation of lipid in 3T3-L1 cells

**Figure 1:**
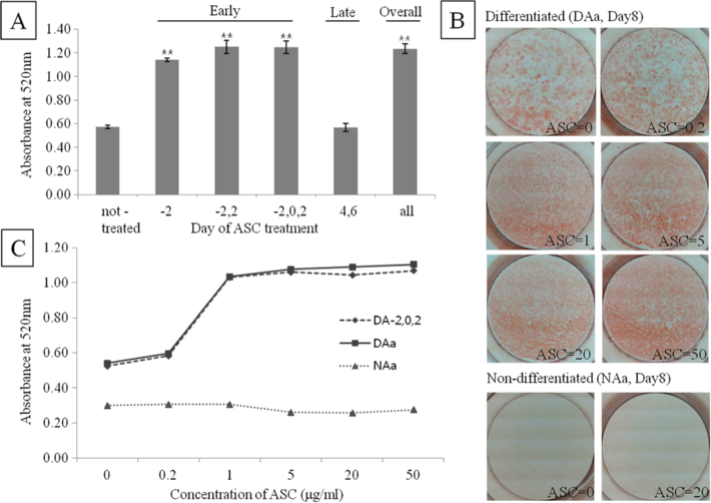
**ORO staining in 3T3-L1 cells was increased by ASC. (A)** Effective phase of ASC treatment was determined by ORO staining on Day 8 after chemical induction. Numbers on x-axis are the day when ASC was treated. -, non-treated; all, ASC treated in overall phase. Data are expressed as means ± SEM (bars). **p < 0.01 vs not-treated. **(B)** Pictures were taken on Day 8 after chemical induction (Differentiated) or not (Non-differentiated). Various concentrations (0-50 μg/ml) of ASC were added on overall phase. **(C)** Effect of various ASC concentration was determined by ORO staining on Day 8 after chemical induction (DA_-2,0,2_ for early phase and DAa for overall phase) or not (NAa for overall phase).

To show the effect of ASC on adipogenesis, ASC (50 μg/ml) was added to chemically induced mouse fibroblast every other day. When 3T3-L1 cells were differentiated into adipocytes up to 8 days after induction (Day8), lipid began to be accumulated from Day4 and reached almost plateau on Day8 (Data not shown). Compared with control cells, lipid was accumulated twice in cells ASC-treated on early phase, even in cells ASC-treated only once (Figure 1A). However, there is no difference in lipid accumulation between control and ASC-treated on late phase confirming the importance of early phase treatment of ASC. With various concentration of ASC this adipogenic effect was increased in dose-dependent manner to a concentration of 1 μg/ml, and then reached a plateau after this concentration (Figure 1B and C). ASC showed no differentiating ability without adipogenic induction in a short term (8 days) culture (Figure 1B) although long term culture of 3T3-L1 with ASC-phosphate showed lipogenic ability [16].

### ASC modulates processing and expression of collagens

**Figure 2:**
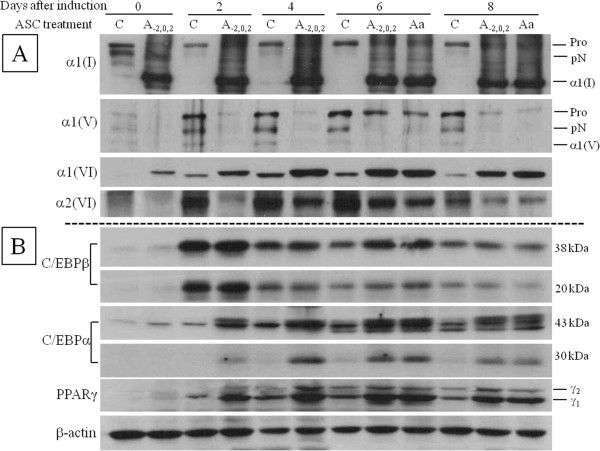
**Expression of collagens and transcription factors was modulated by ASC.** Cell extract of cell layer on Day 0, 2, 4, 6, and 8 was immunoblotted with **(A)** anti-collagen antibodies or **(B)** anti-transcription factor antibodies. ASC was treated early phase (A_-2,0,2_) or overall phase (Aa) or not-treated (control, C). Pro, procollagen; pN, N-procollgen; α1(I), mature α1 chain of type I collagen; α1(V), mature α1 chain of type V collagen.

When ASC was added in early phase of differentiation, procollagen of α1(I) was processed to mature form which was maintained to the late phase with the same degree as ASC treated in overall phase (Figure 2A). Like type I collagen, procollagen of α1(V) also was decreased on addition of ASC although mature α1(V) was not detected because appropriate antibody was not available. Interestingly, the expression of α1 of type VI was increased by adding of ASC while that of α2 of type VI was decreased (Figure 2A). To investigate how these changes of collagen affect the expression of transcription factors of adipogenesis, cell extract was immunoblotted by anti-PPARγ and anti-C/EBPs. C/EBPβ was expressed high after chemical induction and gradually decreased with no difference between ASC treated and control except on Day6. Two major transcription factors, C/EBPα and PPARγ, were increased from Day2, expressed high on Day4 and Day6, and decreased on Day8 in ASC treated (Figure 2B). Unlike collagen expression, the expression of these transcription factors by ASC treatment were not clearly increased before chemical induction, but ASC with chemical induction potentiated the effect on expression of these factors. In control, the expression pattern of these transcription factors was similar to ASC treated but the amount of them was much less than ASC treated, which indicated ASC enhances adipogenesis by intrinsic adipogenic cascade.

### Inhibition of ASC affects the accumulation of lipid droplet

**Figure 3:**
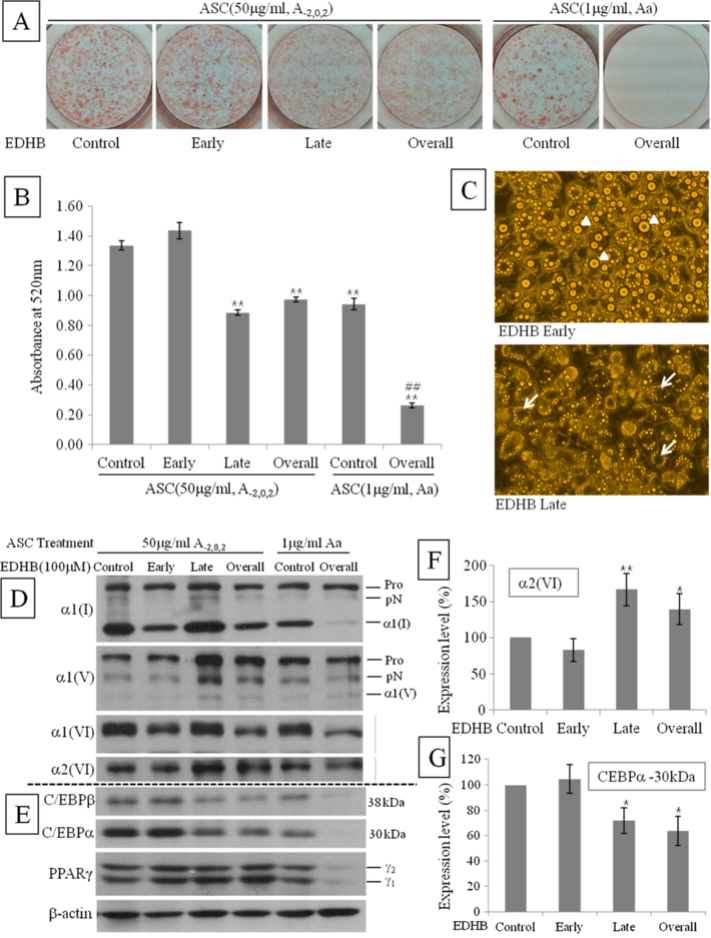
**Inhibition of ASC in late phase removed lipid droplets from adipocytes.** Cells were treated with high concentration (50 μg/ml) of ASC on early phase (A_-2,0,2_) or low concentration (1 μg/ml) of ASC on overall phase (Aa). EDHB (100 uM) was applied to ASC-treated cells on early, late, or overall phase. EDHB non-treated cells were regarded as control. Inhibitory effect was determined by ORO staining on Day 8 after chemical induction. After pictures **(A)** were taken, ORO stain was extracted and absorbance **(B)** was measured at 520 nm. Data are expressed as means ± SEM (bars). **p < 0.01 vs control of high ASC, ##p < 0.01 vs control of low ASC. **(C)** Microscopic determination of inhibitory effect. Arrow heads indicated large lipid droplets while arrows indicated the empty spots of large lipid droplet. **(D)** Immunoblot determination of inhibitory effect on collagens. Pro, procollagen; pN, N-procollgen; α1(I), mature α1 chain of type I collagen; α1(V), mature α1 chain of type V collagen. **(E)** Immunoblot determination of inhibitory effect on transcription factors. Expression level of a2(VI) **(F)** and CEBPa (30 kDa) **(G)** in high concentration of ASC (50 μg/ml, A_-2,0,2_) was semi-quantified. After adjusting control as 100%, band densities are expressed as means ± SEM (bars). *p < 0.05 and **p < 0.01 vs control.

To investigate the effect of ASC inhibition, 100 μM of EDHB, an analogue of ASC, was added on early, late, or overall phase after treating with high concentration of ASC (50 μg/ml, early phase, A_-2,0,2_). EDHB (100 μM) was also added with low concentration of ASC (1 μg/ml, overall phase, Aa). In low concentration of ASC, EDHB completely inhibited adipogenesis (Figure 3A, compared with Figure 1B non-differentiated), reduced expression and processing of collagens, and inhibited expression of transcription factors (Figure 3D and E). This observation indicates that inhibition of collagen synthesis could prevent preadipocytes from adipogenic differentiation in spite of chemical adipogenic induction. In contrast, in high concentration of ASC, ORO staining was lessened in the adipocytes treated with EDHB in late or overall phase whereas adipocytes treated with EDHB in early phase was stained same as control (Figure 3A and B). It suggested that 100 μM EDHB could not inhibit the action of high concentration ASC in early phase of adipogenesis. Intriguingly, ORO staining of late or overall phase was less clear than that of control or early phase, which is explained by the microscopical observation that large lipid droplets was removed from the differentiated adipocyte (Figure 3A and C). In immunoblot experiments on Day8, αl(V) and α2(VI) collagen were increased not in early EDHB-treated but in late EDHB-treated (Figure 3D and F, Late and Overall). This tendency is different from α1(I) and α1(VI) collagens which were increased in ASC-treated on early phase without EDHB (Figure 3D, Control and Late). For transcription factors, C/EBPs were decreased in the groups where α1(V) and α2(VI) were increased (Figure 3E and G, Late and Overall), but PPARγ was not different in all four groups (Figure 3E).

### Inhibition of lipid accumulation may be caused by alteration of surface collagens

**Figure 4:**
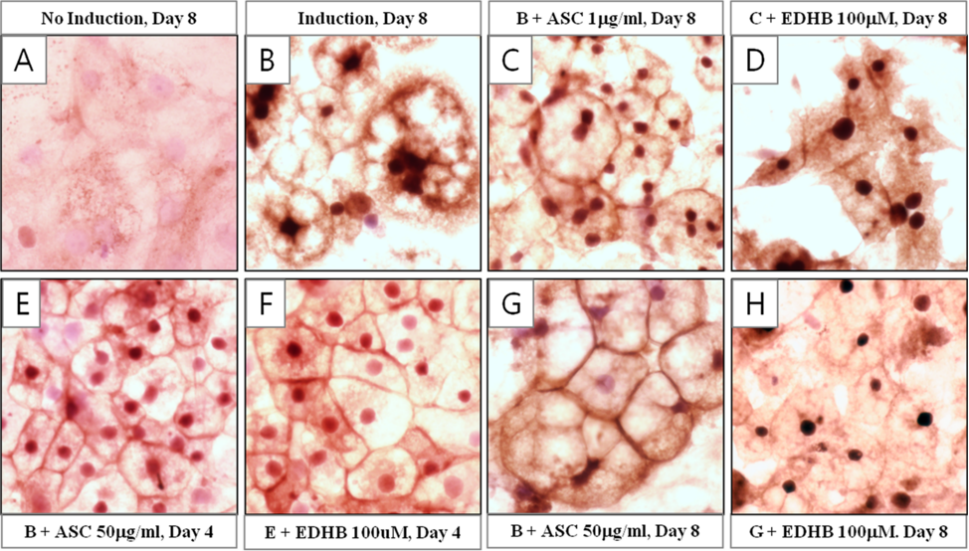
**Inhibition of ASC on late phase caused alteration of type I collagen on adipocyte surface.** Immunostaining analysis was performed on the cells cultured with chemical induction **(B)**, chemical induction plus low concentration (1 μg/ml, Overall phase) of ASC (**C** and **D**), chemical induction plus high concentration (50 μg/ml, Early phase) of ASC (E – H) and without chemical induction **(A)**. EDHB was added on early phase **(F)** and overall phase (**D** and **H**). Pictures were taken on Day 8 (**A** – **D**, **G**, and **H**) and Day 4 (**E** and **F**).

To gain further insights into the nature of inhibition of lipid accumulation, immunostaining with anti-type I collagen was performed on the 3T3-L1 cells treated by ASC and EDHB. Without chemical induction, the staining of type I collagen in cells was not clear and small dots were only seen in some area (Figure 4A). On 8 days after induction (Day8), cells containing lipid droplets were seen apparently and staining of type I collagen in cell margin was more diffuse than in ASC treated cells after induction (Figure 4B, C, and G). Addition of ASC inhibitor decreased the staining of cell margin but increased intracellular staining of type I collagen in low concentration of ASC (Figure 4D). To compare effect of early and late inhibition, cells were stained on Day4 for early inhibition and Day8 for late. Staining on Day4 was similar between EDHB-treated and ASC control (Figure 4E and F) whereas staining in cell margin on Day8 was quite different between EDHB-treated and ASC control as above mentioned (Figure 4G and H). Combined with the results of collagen immunoblot in Figure 3D, this finding proposes the possibility that late EDHB treatment may disturb the fibrillar network on the surface of adipocytes by modulating collagen deposition.

## Discussion

As regenerative technology with stem cells are progressively developed, ASC are used as a cofactor of differentiation from stem cells into several lineages. However, in adipocyte differentiation ASC is not a common additive to induce and maintain the adipogenesis [21] although some papers have presented that ASC enhanced adipogenesis and was related to lipid accumulation through expression of specific collagens [17,19]. Recently, due to its ability of ECM components secretion, ASC is also used for the construction of autologous adipose substitute, which is made from adipose-derived stem cells [22,23]. Despite potency of ASC in adipogenesis, little is known about its diverse effect on deposition of individual collagens in ECM and their complex interaction during adipogenesis.

In our study, consistent with previous reports, ASC enhanced the accumulation of neutral lipid in adipogenesis of murine preadipocyte 3T3-L1 cells with up-regulation of transcription factors, PPARγ and C/EBPs. Generally, in the early phase of adipogenesis C/EBPβ induces expression of major transcription factors, C/EBPα and PPARγ, which mutually induce the expression of each other and cooperate in the adipogenic process. Herein, the effect of ASC treatment on the expression of transcription factors was delayed until chemical induction while the effect on collagen modulation was apparently detected before chemical induction. This time lag indicates that ASC induces ECM remodeling first by change of collagen deposition, which enhances the expression of transcription factors. This observation is supported by previous paper in which fibroblastic preadipocyte was transformed into spherical adipocyte during adipogenesis accompanied by increased flexibility of ECM and alteration in connection among collagens. This change of cell-ECM organization may cause sequential coordination of transcription factors such as PPARγ or C/EBPs [24].

Compared with transcription factors, pattern of collagen expression was more complicated. The bands of two fibrillar collagens, pro-α1(I) and pro-α1(V), were disappeared by ASC treatment confirming that ASC makes these procollagens formed and stable, and helps them be secreted easily to ECM in which the secreted procollagen can be processed to mature collagen by N- or C-proteinase. In addition, it was reported that ASC increased the transcriptional level of C-proteinase enhancer and activity of procollagen C-proteinase in rat fibroblast [25]. From above, we can deduce that decrease of procollagen α1(I) and α1(V) in our result was caused by indirect and direct effect of ASC on collagen processing. We proved it by showing the increase of mature α1(I). However, we could not detect mature form of processed α1(V) because unfortunately appropriate antibody was not available. Usually, collagen antibodies are produced against C-terminal or N-terminal propeptide which is cleaved and does not exist in mature form. Due to this methodological limit, although we didn’t detect the increase of mature α1(V) with anti-α1(V) antibody produced against N-terminal propeptide of α1(V), we considered the loss of pro-α1(V) as processed, not as reduced.

Contrary to fibrillar collagens, α1 and α2 of type VI non-fibrillar collagen was shown in one band and they were expressed in same pattern during adipogenesis. When cells were treated with ASC, ASC increased the expression of alpha1 but decreased that of alpha 2 (Figure 2A), which might be related to high degree of adipogenesis by ASC treatment. Type VI collagen is a nonfibrillar collagen connecting cell surface proteins to ECM components [26] and is the most abundant collagen in major adipose tissues in wild type mice. In recent research, ablation of type VI collagen in obese mice showed that cell size of epididymal adipose tissue was increased while accumulation of hepatic lipid was significantly decreased compared to obese control mice [27]. Considering this conflict between cell size and lipid accumulation, it is suggested that differential expression of α1(VI) and α2(VI) in our study enhance morphological change and lipid accumulation.

ASC activity in collagen biosynthesis has been prevented by its structural analogs. Among these analogs, EDHB competitively inhibits prolyl 4-hydroxylase during production of procollagen [28,29]. Surprisingly, when the action of ASC on collagen regulation was inhibited by EDHB in late phase of adipogenesis, accumulated lipid droplet was removed from fully differentiated adipocytes. (Figure 3C) Because we also observed floating lipid droplets in culture media under microscopic observation, we can assure that the huge empty spots inside EDHB treated adipocytes are the room for lipid droplets left. This observation promptly elicits the hypothesis that changes of fibrillar network on adipocyte surface might allow lipid droplets to leave the adipocytes. This hypothesis is supported by immunostaining result that depositon of type I collagen was decreased on adipocyte surface by addition of EDHB. This phenomenon took place concomitantly with increase of α1(V) and α2(VI) and decrease of C/EBPs (Figure 3D - G). Relationship between increase of both collagens and alteration of fibrillar network can be elucidated by the fact that type V collagen regulates the size of type I collagen fibril and type VI collagen connects surface fibrils to ECM components. Type V collagen is a quantitatively minor fibrillar collagen which is inserted into type I collagen fibril and regulates the diameter of type I/V heterotypic fibrils inversely [30]. Thus, its up-regulation is supposed to participate in reduction of type I collagen on adipocyte surface. Furthermore, collagens synthesized and secreted during adipogenesis are distributed between ECM and adipocyte forming fibrillar network on surface of adipocyte [7,31]. Besides type IV collagen, type I, III, V, and VI collagens are interconnected and forms pericellular fibril structure. Among them, type I collagen was deposited on surface of mature adipocyte [32] and type VI collagen null mouse also showed structural changes in the surface of epididymal adipocytes [27]. Combined above previous papers, overexpression of α2(VI), a marker of preadipose state, can help the interpretation of loss of type I collagen by altering interconnection between surface collagens.

Intriguingly, addition of EDHB on late phase lessened the expression of C/EBPs while did not affect the expression of PPARγ. Although lipid accumulation during adipogenesis in C/EBPα knock-out mouse embryonic fibroblasts was inhibited, exogenous PPARγ recovered its inhibition. However, the size of accumulated lipid inside PPARγ induced cells was larger than C/EBPα knock-out cells [33]. It is suggested that reduced C/EBPs and normal PPARγ in EDHB treated cells participate in regulating lipid accumulation.

## Conclusions

The present study confirmed potentiating effect of ASC on adipogenesis through enhancing the intrinsic adipogenic process, which enforces a possibility of ASC to use in production of adipose substitute from stem cells despite of species specificity for ASC biosynthesis between mouse and human. In addition, inhibitory effect on lipid accumulation by ASC antagonizing showed the plausible way to reduce obesity although our results did not reveal direct mechanism of removal of lipid droplets.

## Materials and methods

### Cell culture and differentiation condition

3T3-L1 mouse embryo fibroblasts cells (3T3-L1) were purchased from Korean Cell Line Bank (KCLB, Seoul, Korea). Cells were cultured in Dulbecco’s modified eagles medium (DMEM) supplemented with 10% bovine calf serum (BCS) for maintenance and cultured in DMEM with 10% fetal bovine serum (FBS) from chemical adipogenic induction on. In order to produce mature adipocytes, 6.7×10^4^ cells of 3T3-L1 were seeded on 6 well plate with DMEM-BCS and cultured for 3 days to be 100% confluent. The day on which cells reached confluency was referred to Day-2 and cells were treated with ASC and/or EDHB (100 μM) from this day on. After grown 2 more days in BCS media (Day0), cells were induced to be differentiated to adipocytes for 2 days with DMEM-FBS containing 1 μM dexamethasone and 250 μM 3-isobutyl-1-methylxanthine. On Day2, cells were refreshed with DMEM-FBS containing insulin (10 μg/ml) and media was replaced with DMEM-FBS every other day. In this study, treatment of ASC and EDHB was performed in two separate phase, early phase of Day-2, Day0, and Day2, and late phase of Day4 and Day6 with low concentration (1 μg/ml) and high concentration (50 μg/ml) of ASC. Induction chemicals, ASC and EDHB were purchased from Sigma Chemicals (MO, USA). Pictures were taken by Nikon Eclipse inverted microscope.

### Oil Red O staining

To determine the degree of differentiation, cells were stained with Oil Red O (ORO, Sigma Chemicals) on the eighth day after the induction of differentiation according to Kasturi and Joshi [34]. Briefly, cells were fixed with 4% paraformaldehyde overnight and washed with 60% isopropanol. After drying cells, 0.21% ORO in 60% isopropanol was applied to the cells for 10 minute followed by 4 times washing with distilled water. Stained ORO was extracted with 100% isopropanol and absorbance was measured at 520 nm. Pictures were taken before extraction.

### Immunoblotting

On the designated day, cells were extracted with 5x Laemmli buffer and 5% *β*-mercaptoethanol, boiling for 10 min. The samples were separated on SDS–polyacrylamide gel and then the gel was transferred to nitrocellulose membranes. Non-specific binding sites on the membranes were blocked in 5% non-fat dry milk for 90 min at RT and membrane was blotted with primary antibody at 4°C overnight and secondary antibody for 90 min at RT. Blots were visualized using the chemiluminescence kit (Immunocruz, Santa Cruz Biotechnology, CA, USA). Primary antibodies for collagens are rabbit polyclonal antibody LF68 (generous gift from Dr. Larry Fisher, NIDCR, NIH) against carboxy-telopeptide of α1(I) collagen, rabbit polyclonal antibodies against extracellular domain of α1(V) collagen or α2(VI) collagen, and mouse monoclonal antibody against α1(VI) collagen. Rabbit polyclonal antibodies against C/EBPα or β and mouse monoclonal antibody to PPARγ were used for adipocyte differentiation regulating proteins. Mouse monoclonal antibody to β-actin was used for loading control. All antibodies for immunoblot were purchased from Santa Cruz Biotechnology.

### Immunostaining

3T3-L1 cells were grown on glass coverslips to the designated day at 37°C in 6-well tissue culture plates. After removal of media, cells were washed in PBS for 5 min and then fixed in 4% paraformaldehyde overnight. Cells were then washed twice with PBS for 15 min and blocked with blocking solution (2% normal rabbit serum in PBS) for 30 min followed by the addition of rabbit polyclonal antibody against collagen type I (Rockland Immunochemicals Inc, PA, USA) in blocking solution (1:200) and incubation for 2 h at room temperature. After rinsing cells twice with PBS for 5 min at room temperature, cells were treated with biotinylated goat anti-rabbit IgG (H + L) secondary antibody (Vector Laboratories, CA, USA) in PBS (1:400) for 30 min at room temperature. Coverslips were washed 3 times with PBS for 10 min and antigen-antibody complexes were detected using an avidin-biotin complex detection system (Vectastain ABC Kit, Vector Laboratories). Coverslips were stained with DAB Substrate kit (Vector Laboratories), rinsed in water, briefly counter stained with hematoxylin and washed again in water. After mounted on glass slides, coverslips were examined with an Olympus BX51 microscope. Pictures were captured and controlled in Olympus DP72 and DP2-BSW.

### Statistical analysis

All experiments were performed at least 3 times and data were analyzed by Student’s t-test. A value of p < 0.05 was considered statistically significant.

## Abbreviations

ASC: Ascorbic acid; C/EBPs: CCAAT-enhancer-binding proteins; EDHB: ethyl-3,4-dihydroxybenzoate; PPARγ: peroxisome proliferator-activated receptor γ; ORO: Oil Red O.

## Competing interests

The authors declare that they have no competing interests.

## Authors’ contributions

BK made a substantial contribution to the conception and design of this study by performing the experiments, assembling, analyzing, and interpreting the data and drafting the manuscript. KC and HR participated in the experimental work and collected, assembled, and analyzed the data. ML contributed to planning the experiments, discussing the results, and preparing the manuscript. All of the authors have read and approved of the final manuscript.
